# Level of quality management in the Municipal Sports Services, contrast trough EFQM Excellence Model

**DOI:** 10.1186/s40064-016-3549-7

**Published:** 2016-10-22

**Authors:** Alfonso Martínez-Moreno, Arturo Díaz Suárez

**Affiliations:** Faculty of Sport Sciences, University of Murcia, C/. Argentina s/n. Santiago de la Ribera, 30720 San Javier, Murcia Spain

**Keywords:** EFQM, Municipalities, Public services, Quality management, Productivity, Government

## Abstract

The quality management in the Municipal Sports Services is embedded in the servuction provided to the citizens, which are their internal customers who determine the quality improvement ensuring competitiveness with excellence criteria. The Model of the European Foundation for Quality Management enables the evaluation of organization progress towards achieving quality goals, from a structured, measurable and comparable methodology. The aim is to carry out a diagnosis of the level of implementation of quality in the Municipal Sports Services of the Region of Murcia, Spain. The sample of 287 workers of 30 sports services gets a high level of reliability at all scales, with a coefficient of variation of .985 (range .810–.943). The score in the criteria of Policy and Strategy, People Management, Alliances and Resources, Processes and People Results were significantly higher (p < .05) in the Municipalities with more than 25,000 inhabitants when compared with those less than 10,000 and with those from 10,000 to 25,000 inhabitants obtaining global ratings of 571 points, those less than 10,000, 590 points those from 10,000 to 25,000 and those higher than 25,000 reach 636, having a good level of quality in relation to the scale that determines the model.

## Background

The concept of Quality and Quality Management have been adapted over the years, expanding objectives, varying their orientation, updating and adapting quickly to technological changes, of organizations, markets and social demands. It acquires, therefore an increasing importance happening at the beginning from a mere control or inspection to become one of the mainstays in the strategies of public and private organizations. The Quality is the condition *sine qua non* on which should pivot all actions of the Public Administration to provide them with trust, relevance, mobility, compatibility and attractiveness, ensuring processes mechanisms for evaluation, certification and accreditation. The Quality Management will allow differentiating and competing in a new stage characterized by quick changes in supply and demand (Casadesús et al. 2010). The Quality Management is a way of managing activities to gain efficiency, effectiveness and competitiveness, ensuring long-term possibility for the organization to subsist, meeting the needs of its customers, employees, shareholders and society in general (Zargar et al. 2011).

There are few studies of the service quality and user satisfaction of sports entities in the literature related to sport management (Tsitskari et al. 2006), hence the purpose of this study is to analyze the service quality of sports entities in the Region of Murcia. In the specialized literature, there are relatively few studies from the perspective of the services provider, some have analysed the management and policies carried out by the Municipal Sports Services (SDM), (Gharakhania et al. 2011). There are studies on the service quality of sport entities services focused on the evaluation of expectations, satisfaction and perceptions of quality by users, using among other scales: QUESC (Quality Excellence of Sports Centres) (Kim and Kim 1995); NEPTUNO 1 (Luna et al. 1998), FITSSQ (Fitness and Sport Service Quality) (Papadimitriou and Karteroliotis 2000); inventario de Calidad en Programas de Actividad Física (I.C.P.A.F.), (Hernández 2001); SERVQUAL (Barrera and Reyes 2003; Calabuig et al. 2010; Dorado and Gallardo 2003; Mañas Giménez et al. 2008; Morales Sánchez et al. 2004; Morales et al. 2005; Morales et al. 2009; Morales Sánchez 2003; Salvador 2005) QSport-10 (Rial-Boubeta et al. 2010), and more recently the DEPQUAL scale (Salazar 2015) that measures the quality perceived by athletes and users in events and sports facilities, most of them with little market penetration. Different approaches to analyse the management of municipalities have been used in other studies, some through the analysis of municipal sports policies and its evaluation (Redondo 1997), others analysing its functions and manifestations in management (Martínez del Castillo 1994) and analysing user satisfaction (Dorado 2006).

Regardless of the sector, size, structure or maturity, the organizations need to establish appropriate management systems. The performance improvement of the public sector is a priority strategy in OECD countries (OECD/INAP 2006; OECD 2011). The service quality has been recognized as a key factor obtaining competitive advantages and, in particular, for customer retention (Ching-Chow et al. 2011). Many researchers believe that Total Quality Management promotes competitiveness by establishing a continuous improvement in the different areas that make up the company; both what we call internal quality, activities improvement (Mak 2011; Lee et al. 2009; Sila 2007), and external quality or performance of firms (Powell 1995; Kaynak 2003).

For the diagnosis of quality to have a strategic projection, the selected model should not be about minimums, but an explicit, structured, validated, recognized model of excellence with extensive practical application and, as far as possible, with effective and international prestige. It has been opted for the application of the *European Foundation for Quality Management* (EFQM) of Excellence Model, contextualized to the field of sports services with the questionnaire of Quality Municipal Services (SERMUCAL) Martínez-Moreno et al. (2006) that offers to SDM, a practical tool to address evaluation systems and measurement in its path to excellence, helping them understand their shortcomings and to stimulate the search for solutions. It allows to analyse and evaluate an organization in each of the key aspects: (a) to identify its strengths and areas for improvement, (b) to establish a level of excellence (score) in each of the key aspects, (c) to establish priorities on where to act. Araújo and Sampaio (2014) showed how organizations that have achieved an EFQM recognition have progressed through a more mature development process and advanced stages. There is a clear predominance of the use of this model in countries with disparate structure of markets and institutional environment, such as UK, Spain, Germany, Italy and Turkey (Allur 2010). The application of the principles of excellence, according to the EFQM model is a very popular tool and accepted positively to achieve continuous improvement (Petrič and Gomišček 2011). Given that workers play a major role in increasing productivity in any organization, but even more in services (Beikzad et al. 2011).

Most of the studies have been done from the standpoint of measuring the service quality and not on quality management, there are hardly studies focusing on internal processes and how they are managed and developed by internal-workers customers, in order to offer a quality service.

From the foregoing, we raised this empirical study by the vacuum detected in the field of Quality Management in the field of Municipal Sports Services. The purpose of the study is to carry out a diagnosis of the level of implementation of quality within the SDM, differentiating them by the number of less than 10,000 inhabitants, between 10,000 and 25,000 and 25,000. The hypothesis is that the quality will be better in the third group (25,000). The workers (internal customers) are those who, through self-evaluation offer their perspective of needs and strengths, under the paradigm of the EFQM model, providing a common analysis methodology to compare results.

### Sport: organization and evolution

Public Administration has three levels and according to them the sports organization is established: (1) Central administration, called General Administration of the State. (2) Regional Administrations, which have an identical organization in all the Autonomous Communities, but the existing models of administrative organization are very similar in all of them. (3) Administrations of local authorities (municipalities, provincial councils and town councils), these usually have an autonomous body to take over the management of the sports area. It is usually referred to as Municipal Sports Office/Municipal Sports Institute/Foundation, among others. It is not often that management is assumed directly through sections or departments in municipalities with more than 25,000 inhabitants. Lately pressured by the brutal economic recession and cuts syndrome, these autonomous entities are disappearing in a dizzying way; to the detriment of the advantages in the field of sports services; requiring immediate answers to perform adequate service quality.

Given the complexity that has reached the local sports system, it is essential to give priority to the quality of these services in its entirety. To be able to reach it, all internal customers (cleaning and maintenance personal, monitors, sports technicians, coordinators, managers, etc.) are of central importance in the final result of quality (Pérez-Arechaederra et al. 2010).

Municipalities are the most dynamic organizations that perform a function of promotion and development of sports practice and are the greater managers of sport in the public sector (Delgado 2000). As the Municipalities are the closest Public Administration to citizens, it has to be alert to their demands having to provide fast, effective and efficient solutions favoured by a Quality System. Determine a quality criterion is essential for proper optimization between users and organization and positively influence the loyalty of users (Gálvez and Morales 2011).

### The EFQM model of quality excellence

The European *Foundation for Quality Management* is a non-profit organization founded in 1988 by 14 multinational European companies, with the aim of achieving sustainable excellence. It was updated in 1999, 2003, 2010 and 2013 with greater orientation towards economic and social sustainability (EFQM 2010) version on which is based the present study.

The EFQM model of quality excellence, for its globalist character, covers all aspects of the functioning of an organization and allows a comprehensive approach to all development processes and at all levels of its structure. It is a non-prescriptive framework based on nine criteria (each one divided in different sub-criteria) and with a certain specific weight, modified in the last update of 2012, related to all areas of management and measurement of organizations results. Five criteria are agents (leadership, policy and strategy, people, alliances and resources, processes) and four additional criteria are the results (customers results, workers results, community results, key performance results); agents “criteria that cover what is done by the organization and the criteria of” results “, covering the achievement of an organization (Sadikoglu and Olcay 2014).

## Methods

### Population and sample

The population studied comprises the total of direct and indirect workers that enable and perform the public offering of the SDM in the Region of Murcia. The formula for infinite population, with a level of confidence of (95.5 %), an error of 5.5 %, p = 50 % and q = 50 % (Sierra 1988) was used to determine the optimal minimum sample size. When applying the formula, a minimum number of 267 workers was calculated.

It was based on the model developed by the EFQM (European Foundation for Quality Management 2010), which consists of nine factors. Each variable was included in a single factor, depending on its factorial load, setting values of30 as a minimum saturation criterion (Ferrando and Anguiano-Carrasco 2010).

The final sample consisted of 287 workers, 172 (50.9 %) men and 115 (40.1 %) women, of all groups and professional levels of local Administration (cleaning and maintenance personal, office clerks, administrative assistants, sports technicians, coordinators, managers, etc.) except for political office, of 30 SDM of the Region of Murcia. Three categories of municipalities depending on the number of registered inhabitants were defined: (1) municipalities with less than 10,000 inhabitants (A); (2) municipalities with a population between 10,000 and 25,000 inhabitants (B); (3) municipalities with more than 25,000 inhabitants (C).

Out of a total of 30 municipalities analysed, the final distribution of the sample was: A-10 municipalities (i.e. 23.0 %), B-11 municipalities (i.e. 30.7 %) and C-9 municipalities (i.e. 46.3 %). Type A municipalities had at least a football field, a pavilion and a winter and/or or summer swimming pool. The type B municipalities had two football fields, two pavilions, an indoor and/or summer swimming pool, and type C municipalities had at least more than three football fields, more than three pavilions and more than three indoor and/or summer swimming pools. The types of programs were similar in the different municipalities, varying the number of them, ranging from five to ten programs by installation.

### Instrument, construction, implementation and punctuation

The data were obtained using as a basis the questionnaire of Quality Municipal Services (SERMUCAL) (Martínez-Moreno et al. 2006), following the self-assessment methodology of the EFQM model covering the criteria and sub-criteria defined by the model, contextualizing the language thereof to the SDM.

The questionnaire consists of a total of 116 items, of which eleven are socio-demographic (i.e. municipality, gender, age, educational qualifications, completed studies, job title or position performed, group and level, name of the agency or entity that manages sport in the municipality, form of access to the workplace, employment status, functions performed in the Department-Service-Board-Municipal Institute, length of service, seniority in the Department-Service-Board-Municipal Institute). The remaining items are closed answers with a Likert scale ranging from 1 Nothing or very little, to 4. Being distributed as follows: Criterion (1) Leadership (11 items), (2) Policy and Strategy (12 items), (3) People management (11 items), (4) Alliances and Resources (12 items), (5) Processes (11 items), (6) Results for external users-customers (14 items), (7) People results (12 items), (8) Results in society (7 items), (9) Key results (15 items).

### Statistical analysis

The cognitive pre-test was carried out by ten experts (Dunn et al. 1999), defining as expert a Doctor of Science of Physical Activity and Sport and/or of Economics, with a minimum experience of 10 years in the field of sports management. The experts were selected to adequate the language of the EFQM model to the context of sport municipal services.

An intentional non-probabilistic sampling was used as evaluation of the psychometric properties of the questionnaire, the content validity, criterion and construct and internal consistency were analysed by a pilot study. For calculating the content validity, each expert judge was asked, in each of the items of the EFQM, the level of understanding, difficulty and length of the questionnaire. For the content validity, the V Aiken test was carried out (Penfield and Giacobbi 2004), an adaptation of the dimensions .95; a balance of the dimensions .92 and enough specification of content through the set of items .97. Adjustments in the wording of items .92; clarity in the wording .96 and importance of the items .95.

The characteristics of the sample were studied by frequencies, percentages, means and standard deviation (SD) to compare the values of different EFQM model criteria, between different samples depending on the size of the municipality (A, B, and C). The parametric one-way ANOVA test was used, with post hoc Sheffe to construct validity with the confirmatory factor analysis (Thomsom 2004). Since the number of variables is very high, the unweighted least squares method (ULS) was used. The RMSEA (root-mean-square error of aproximation) was used as statistician setting, the goodness of fit index (GFI). The total variance explained that the nine factors account for a (71.38 %) percentage of explanation which stands at very high levels of acceptance. Specifically, the management people factor explains a (22.01 %) of the variance, the leadership factor a (8.35 %), the key results factor a (7.26 %), the results in people factor a (6.44 %), the external customers results factor a (5.84 %), the policy and strategy factor a (5.67 %), the results in society factor a (5.45 %), the alliances and resources factor a (5.25 %) and the processes factor a (5.11 %). The Cronbach’s Alpha was used for the reliability of the EFQM model, indexes located around 70 suggest that there is an adequate internal consistency (Celina and Campo 2005; Ferrando and Anguiano-Carrasco 2010). It can be observed in the next table (Table 1).

**Table 1:** Reliability: internal consistency of EFQM

Factors	Cronbach’s alpha
People management factor	.903
Leadership factor	.939
Key results factor	.925
Results in people factor	.888
External customer results factor	.897
Policy and strategy factor	.917
Results in society factor	.810
Alliance and resources factor	.918
Processes factor	.943
EFQM	.985

The subscales are with higher values than .800, with an average of .985 (Range .810–.943) in the total scales of the Model.

The data analysis was performed using the statistic programme SPSS 19.0, in its version for Windows. The statistical analysis was performed with a significance level of p ≤ .05. The factorial confirmatory analysis was performed with the program, LISREL version 8.54.

## Results

The score in the Policy and Strategy, People Management, Alliances and Resources, Processes and Results in people criteria were significantly higher (p < .05) in the municipalities C, when compared with the results obtained in the municipalities A. When the results obtained by the municipalities C and B were compared, the latter had significantly lower values (p < .05) in the criteria for Policy and Strategy, Management of People, Alliances and Resources and Processes. The rest of the criteria (i.e. Leadership, External Customers Results, Results in Society, Key Results) showed no significant differences when comparing the different types of municipality (Table 2).

**Table 2:** Average scores (points and %) obtained according to the number of inhabitants

	Municipalities A		Municipalities B		Municipalities C	
	Points	Means ± SD	Points	Means ± SD	Points	Means ± SD
Leadership	626	62.6 ± 23.1	666	66.6 ± 22.9	690	69.0 ± 19.4
Policy and strategy	506	50.6 ± 21.2	528	52.8 ± 19.5	606	60.6 ± 19.9*,^#^
Person management	551	55.1 ± 18.7	558	55.8 ± 19.3	629	62.9 ± 18.8*,^#^
Alliances and resources	502	50.2 ± 19.8	528	52.8 ± 20.4	624	62.4 ± 19.3*,^#^
Processes	514	51.4 ± 24.6	544	54.4 ± 20.2	615	61.5 ± 20.7^#^
External customers results	627	62.7 ± 18.9	637	63.7 ± 16.0	657	65.7 ± 14.7
Results in people	602	60.2 ± 17.7.	576	57.6 ± 16.3	640	64.0 ± 15.3*
Results in society	569	56.9 ± 18.5	594	59.4 ± 14.7	621	62.1 ± 16.4
Key results	644	64.4 ± 17.2	675	67.5 ± 14.9	676	67.6 ± 15.3

* Statistically significant differences between municipalities C and A

^#^ Statistically significant differences between municipalities C and B

**Fig. 1:**
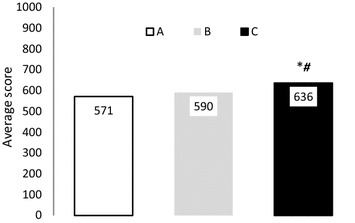
EFQM model of total scores by type of municipalities

For its part, the results of the total score of the different scales that make up the EFQM model show significantly higher values in the municipalities C, when compared with the results obtained by the municipalities A and B (p < .05) (Fig. 1).

## Discussion

All studies consulted are located in external users-customers, so for the authors’ knowledge, this is the first study that has carried out a comprehensive and strategic analysis to determine the degree in quality management by internal customers of the SDM, applying the EFQM model with a large sample (i.e. 30 sports services in total). Despite this, few researchers have obtained results with the application of the EFQM model, of Rial et al. (2004) in the sports service of the University of Vigo onwards SDUV (Spain) and Galán (2004) in the sports activities service of the University of Seville onwards SADUS (Spain).

The main findings of the study indicate that sports services of the municipalities A and B, according to their internal customers, reach values over 50 % and the municipalities C, over 60 % of the total indicated in the model, reveal the existence of a generally positive assessment by internal customers.

In relation to individual analysis of each of the criteria, leadership, external customer results, results in society and key results showed no significant differences when comparing the type of municipality. Reaching the three types (A, B and C) values higher than 62.0 % in the criterion of Leadership, so they have all the commitment of leaders. Numerous studies analyse the importance of the commitment of the leaders in achieving improvements and reach the total quality (Akdere 2009; Escrig-Tena and Bou-Llusar 2005; Escrig-Tena et al. 2001; Roca-Puig et al. 2006).

The three groups of SDM exceed 50 % of the total tested by the model, as the criterion Policy and Strategy with very superior values to those from Rial et al. 2004 (42.3 %), Galán 2004 (45.0 %). Services C achieve a (60.6 %), however this criterion is still the least valued by internal customers of these Sports Services.

Regarding the People Management, these results range from (55.1 to 62.9 %), and indicate that the SDM begin to consolidate it as it is a basic pillar for the success of quality management, the improvement is an evolution of organizational learning, providing the participation of all people in improvement activities rewarding and recognizing their efforts. The SDM, A and B obtain similar values (55.1 %) and (55.8 %) respectively, quadrupling the (12.0 %) obtained by Rial et al. (2004) and above the (31.0 %) of Galán (2004). Services C surpass all the previous (62.9 %).

As to Alliances and Resources, Services A (50.2 %) and B (52.8 %) obtained values in the line of Galán (2004) (47.0 %), exceeding the (30.9 %) of Rial et al. (2004), Services C get a (62.4 %) standing out above other services.

It is imperative to have a system of indicators to evaluate the effectiveness and efficiency of Processes, which can be performed through periodic surveys, suggestion- complaints box. The SDM, A and B obtain similar values (51.4 %) and (54.4 %) far from Rial et al. (2004) (28.7 %) and a (40.0 %) achieved by Galán (2004), Services C, arrive at a (61.5 %),

About the External Customers results, the data achieved by the service A (62.7 %), B (63.7 %) and C (65.7 %), are almost three times higher than those of Galán (2004) with a (15.0 %) and widely exceeded the (22.2 %) obtained by Rial et al. (2004). So the key processes arise from the needs, interests and preferences of users and a good relationship with them to achieve good results countersigned by various authors (Henning-Thurau and Klee 1997; Anderson et al. 1994; Heskett et al. 1994; Lehtinen and Lehtinen 1991; MAP 2006). A proper attention to users is a key element to achieve high levels of quality in management, both in companies in general (Bitner et al. 1994; Price et al. 1995), and in services and sports companies in particular (Carver 1997), key elements to achieve high levels of adherence to the service (Winterstein 1995).

The findings in relation to Management People results the SDM B, obtained the lowest rating (57.6 %), although exceed a (6.6 %) of SDUV (Rial et al. 2004) as well as a (15.0 %) of SADUS (Galán 2004), Services A (60.2 %) above the C, with a (64.0 %) indicating that there are high rates of motivation and satisfaction.

The Results in society are valued in a range between 56.9 and 62.1 %, as the services A and B obtain values below 60 %, suggesting that the services under study comprehensively measure the perception that society has of the organization and how to reach their results. High values are obtained in the relations of the SDM with other sports services and other institutions, and low values in the complaints made by individuals and institutions of the environment, both aspects directly related to high levels of quality (Inglis and Chelladurai 1987).

The values described regarding Key results, the three types of services obtain similar scores A (62.6 %) B (66.6 %) and C (69.0 %) respectively, exceeding (16.5 %) of Rial et al. (2004) and the (30.0 %) achieved by Galán (2004) showing that its economic and financial performance and management volume are optimal.

## Conclusions

The current market horizons, greater competitiveness, strong expansion of corporate globalization, pressure on public and/or private organizations to implement management systems to inexcusably makes the quality its identity mark.

An important aspect that warrants the investigation derives from the importance of self-evaluation processes, increasingly embedded in the daily practice of organizations, which has formed the EFQM Model, as an appropriate tool to analyse and evaluate the organizations under study. The results of this study have important implications for the managers of the SDM, as they provide the strengths and areas for improvement in the field of quality. The application of the EFQM model in its questionnaire version through self-evaluation by internal customers, in the Municipal Sports Services of local authorities, allow to detect the main strengths and weaknesses in order to be effective and efficient in the necessary levelling of the different areas or sections to improve the quality of the studied local authorities, setting the level (score) gained on its way to excellence.

Services A, have in the key results its strength and in the Alliances and Resources its weak point, so that leaders interact with users and social representatives. There appear to be no defined scheme of key processes, besides, there is little dialogue between workers and the organization; however, the planning, management and improvement of human resources are acceptable. However, in the processes, they make innovations to better suit users, for them the results are positive when users want to continue doing activities. There is a good relationship between users and workers; they think that there may be a high impact of their activity in the different municipalities.

Sports Services B have on key results its strength and its weakness in Policy and Strategy and in Alliances and Resources so that their leaders are those that reinforce the culture of excellence, they have well-defined their customers-users, there are rewards and recognition to internal customers but with little or no existence in measuring compliance with the objectives. They argue that there is an adequate attention to complaints, a low rate of absenteeism and good relations with other services.

Services C have in the Leadership its strength, and in the Policy and Strategy its weakness. One of its priorities is the attention to external users-customers, although they materialize that the relationships with suppliers are deficient. They request an extension of the training plan and indicate that the systematic self-evaluation of performance of the duties of workers can be deficient. There is a willingness to interact with other agencies regarding Alliances and Resources. It is important to them that the processes are identified and documented, the teachers of activities are available, they indicate that there is good relationship between internal users-customers, although there are complaints from people and institutions of the environment, the users rate evolves positively.

The management of Municipal Sports Services requests management models that allow to be embedded in the vertiginous dynamics of an increasingly demanding and competitive market, based on criteria of excellence. This model enables reliable feedback, in this study of the internal client itself, allowing detecting, adjusting and redirecting investment of both human and economic capital, and implementing ways to improve service delivery. The EFQM model provides a wonderful opportunity to successfully face the difficult challenges that the organizations and especially sports services are overloking.

One limitation of the study is related to the transversal cut thereof, as this paper has analysed the relationship in a precise moment in time.
